# Association Mapping of Total Carotenoids in Diverse Soybean Genotypes Based on Leaf Extracts and High-Throughput Canopy Spectral Reflectance Measurements

**DOI:** 10.1371/journal.pone.0137213

**Published:** 2015-09-14

**Authors:** Arun Prabhu Dhanapal, Jeffery D. Ray, Shardendu K. Singh, Valerio Hoyos-Villegas, James R. Smith, Larry C. Purcell, C. Andy King, Felix B. Fritschi

**Affiliations:** 1 Division of Plant Sciences, University of Missouri, Columbia, Missouri, United States of America; 2 Crop Genetics Research Unit, USDA-ARS, Stoneville, Mississippi, United States of America; 3 Crop Systems and Global Change Lab, USDA-ARS, Beltsville, Maryland, United States of America; 4 Department of Plant, Soil and Microbial Sciences, Michigan State University, East Lansing, Michigan, United States of America; 5 Department of Crop, Soil, and Environmental Sciences, University of Arkansas, Fayetteville, Arkansas, United States of America; College of Agricultural Sciences, UNITED STATES

## Abstract

Carotenoids are organic pigments that are produced predominantly by photosynthetic organisms and provide antioxidant activity to a wide variety of plants, animals, bacteria, and fungi. The carotenoid biosynthetic pathway is highly conserved in plants and occurs mostly in chromoplasts and chloroplasts. Leaf carotenoids play important photoprotective roles and targeted selection for leaf carotenoids may offer avenues to improve abiotic stress tolerance. A collection of 332 soybean [*Glycine max* (L.) Merr.] genotypes was grown in two years and total leaf carotenoid content was determined using three different methods. The first method was based on extraction and spectrophotometric determination of carotenoid content (eCaro) in leaf tissue, whereas the other two methods were derived from high-throughput canopy spectral reflectance measurements using wavelet transformed reflectance spectra (tCaro) and a spectral reflectance index (iCaro). An association mapping approach was employed using 31,253 single nucleotide polymorphisms (SNPs) to identify SNPs associated with total carotenoid content using a mixed linear model based on data from two growing seasons. A total of 28 SNPs showed a significant association with total carotenoid content in at least one of the three approaches. These 28 SNPs likely tagged 14 putative loci for carotenoid content. Six putative loci were identified using eCaro, five loci with tCaro, and nine loci with iCaro. Three of these putative loci were detected by all three carotenoid determination methods. All but four putative loci were located near a known carotenoid-related gene. These results showed that carotenoid markers can be identified in soybean using extract-based as well as by high-throughput canopy spectral reflectance-based approaches, demonstrating the utility of field-based canopy spectral reflectance phenotypes for association mapping.

## Introduction

Carotenoids are organic pigments that are produced predominantly by photosynthetic organisms and comprise the red, yellow and orange colors of flowers, fruits and other plant organs [1]. In addition to providing color to flowers and fruits they also contribute to the production of scents and flavors that attract insects and animals for pollination and seed dispersal [1, 2]. Carotenoids also provide antioxidant activity to a wide variety of plants, animals, bacteria, and fungi [3]. They are recognized as important health-promoting ingredients in the human diet as some carotenoids have antioxidant properties and may prevent cancer as well as cardiac and eye diseases [4, 5]. The human health benefits associated with carotenoids have been extensively reviewed [6–8].

In plants, carotenoids play important roles in photosynthesis as accessory pigments and in photoprotection. As accessory pigments, carotenoids are involved in light harvesting and energy transfer to chlorophyll [9]. The absorption maxima of carotenoids differ from the absorption maxima of chlorophylls, thus expanding the range of light capture for photosynthesis [10]. The four most abundant carotenoids in leaves are lutein, β-carotene, zeaxanthin and violaxanthin. β-carotenes are found in components of Photosystem I (PSI) and Photosystem II (PSII) complexes where they capture light or photosynthetically active radiation (PAR) [11, 12].

Because unfavorable conditions such as excess light energy can lead to the production of reactive oxygen species that can damage photosynthetic membranes and proteins, light absorption and energy transfer are highly regulated by numerous processes. The essential photoprotective roles of leaf carotenoids include: scavenging of reactive oxygen species, quenching of dangerous triplet states of chlorophyll and participation in thermal dissipation of excess light energy [13]. Carotenoids can rapidly quench excited chlorophylls and thus prevent reactive oxygen species production. Additionally, the three carontenoids, violaxanthin, antheraxanthin, and zeaxanthin (xanthophylls) are also involved in nonphotochemical quenching, which plays a critical role in regulating how much excitation energy is transferred to reaction centers [3, 13, 14]. Carotenoids also serve as precursors for abscisic acid (ABA) and strigolactones [2, 15, 16].

The carotenoid biosynthetic pathway was postulated more than three decades ago based on standard biochemical analyses using labelled precursors, specific inhibitors, and mutant characterization [17]. Genes encoding nearly all of the enzymes involved in this pathway have been cloned from bacteria, fungi, and plants [7, 18, 19]. The carotenoid pathway is highly conserved in plants and photosynthetic bacteria. Carotenoid biosynthesis occurs mostly in chromoplasts and chloroplasts [7]. Typically, leaf tissues contain several carotenoids including lutein, β-carotene, violaxanthin and neoxanthin with changes in their profile altering photosynthesis and photoprotection [20, 21].

In plants, the xanthophyll cycle, the reversible interconversion of two carotenoids, violaxanthin and zeaxanthin with an antheraxanthin intermediate, has a photoprotective role [9, 22]. Extensive investigations of the xanthophyll cycle have clearly demonstrated its role in photoprotection [20, 23, 24] and stress tolerance [25–27]. For instance, manipulation of the xanthophyll cycle pool by overexpression of β-carotene hydroxylase in *Arabidposis thaliana* reduced leaf necrosis and lipid peroxidation and increased tolerance to high light and high temperatures [28]. Other studies have found that increased levels of zeaxanthin increased tolerance to UV radiation and to high light and low temperature in Arabidopsis and tobacco [29, 30]. Because of their nutritional importance, the genetics underlying the accumulation of various carotenoids in tissues used for human consumption has received considerable attention [31, 32], while the genetics underlying leaf carotenoids have not been considered as widely. A recent study using a wheat double haploid population identified 17 quantitative trait loci (QTLs) for leaf carotenoid content [33]. However, despite their importance in photosynthesis and roles in stress tolerance, no studies have reported molecular markers for leaf carotenoid content in soybean.

For quantification, leaf carotenoids are commonly extracted and analyzed by liquid chromatography or spectrophotometer but can also be assessed based on spectral reflectance characteristics of intact leaves [34–36]. Extraction-based analyses are often conducted on small samples that only represent a portion of a leaf, an entire leaf, or a small number of leaves. Reflectance-based methods can be employed for leaf level assessments, often in conjunction with leaf clips, for larger aggregates of leaves or at the canopy level under natural settings [37, 38]. Leaf and canopy-based reflectance methods are receiving much attention for their potential for non-destructive, high-throughput phenotyping under controlled as well as field environments [34, 38–40]. Many studies have associated leaf and/or canopy reflectance characteristics with distinct plant phenotypes and a large number of models and indices have been developed for a range of these phenotypes [10, 37, 38, 41–44]. Since, pigments such as chlorophyll and carotenoids strongly influence light absorption and thus also reflectance, spectral reflectance analysis is a particularly promising approach for quantification of these pigments [34, 36, 45].

To date, no markers for leaf carotenoid content have been reported for soybean. Further, to our knowledge, there are no associations or mapping studies of carotenoid content based on high-throughput canopy spectral reflectance measurements. The objective of this study was to use a genome wide association mapping approach to identify loci associated with one extract-based and two canopy spectral reflectance-based carotenoid content measurements in soybean.

## Materials and Methods

### Ethics statement

No specific permission was required for the field study as it was conducted at the University of Missouri Bradford Research Center. No endangered or protected species were involved in this study.

### Field Experiments and Plant Material

Field experiments were conducted at the Bradford Research Center (BRC) in Columbia, MO USA (38° 53′N, 92° 12′ W) on a Mexico silt loam soil (fine, smectitic, mesic Aeric, Vertic, Epiaqualf). A total of 385 diverse maturity group IV genotypes were planted on 23 May, 2009 and 27 May, 2010 in a randomized complete block design with three replications. Seeds were planted at 2.5 cm depth at a density of 25 seeds m^-2^ in plots that were 4.87 m long and 4 rows wide with 0.76 m row spacing. Standard agronomic practices were employed and carried out as previously described [37]. A subset of 332 genotypes (plant introductions originally obtained from the USDA Germplasm Collection), were included in this study. The 332 genotypes originated from 11 different nations, including 206 from South Korea, 59 from China, 39 from Japan, 11 from North Korea, six from Georgia, four from Korea (North or South Korea not recorded in GRIN), two each from Russia and Taiwan and one each from India, Mexico and Romania. Genotypes were selected based on the USDA Germplasm Resources Information Network (GRIN) data in an attempt to maximize diversity while considering high yields and good agronomic characteristics for one subset (167 genotypes) and geographical origin without consideration of yield but while maintaining good agronomic characteristics such as height, lodging, and shattering for a second subset (165 genotypes) (for additional information on criteria of selection see [46]).

### Carotenoid Determinations

Carotenoid contents were determined by three methods hereafter referred to as extractable carotenoid contents (eCaro), wavelet transformed spectral reflectance carotenoid contents (tCaro), and spectral index carotenoid contents (iCaro). These three carotenoid contents were assessed by 1) spectrophotometric determination in extracts from leaf disks, 2) a spectral reflectance model developed using canopy spectral reflectance measurements and the carotenoid contents determined from the leaf disk extracts [37], and 3) a published spectral reflectance index for carotenoids developed for soybean [10]. Briefly, for spectrophotometric determinations, five leaf disks (0.68 cm^2^ each) were collected from the 3^rd^ or 4^th^ leaf from the stem apex (upper-most fully expanded, sun-exposed leaf) of five different plants per plot at flowering [R1–R2 stage, [47]] in 2009 (54 days after planting; DAP) and 2010 (60 DAP). The five leaf disks were immediately placed into an opaque vial containing 5 mL of ethanol (95%, v/v) and incubated for 24 h in the dark at room temperature. After incubation, vials were vigorously agitated, a 200 μL aliquot was transferred to 96 well-plates (Costech Analytical Technologies Inc., CA USA), and the absorbance was measured at 664, 648, and 470 nm on a Scanning Monochromatic Spectrophotometer (Bio-Tek PowerWave X 340 Microplate Reader, BioTek U.S. VT, USA). Total carotenoid content was calculated according to the equation of Lichtenthaler [48] and expressed on a leaf area basis (μg cm^-2^). This extract-based spectrophotometric carotenoid determination is hereafter referred to as eCaro.

Canopy spectral reflectance was measured using an ASD FieldSpec, FR spectroradiometer (Analytical Spectral Devices Inc., Boulder, CO, USA) between 54 and 57 DAP in 2009 and 58 and 61 DAP in 2010 as previously described [37], and coinciding with the leaf disk sampling. In brief, for each plot, spectral reflectance measurements were collected at three points within each plot with the fiber optic cable positioned about 0.5 m above the plant canopy. The spectrum measured covered the range from 350 to 1800 nm in 1 nm intervals. The three reflectance spectra measurements were averaged, and the spectral reflectance above 1350 nm was excluded because of interference of water bands in this region [34, 37]. The reflectance spectra were then associated with eCaro contents and multiple canopy spectral reflectance-based models were tested for carotenoid content estimation [37]. The model that provided the highest accuracy for carotenoid content estimation was based on multiple linear regression (MLR) analysis and incorporated six wavebands derived from continuous wavelet transformed spectral reflectance data using the ‘Mexican hat’ wavelet family. Thus, this model was used to estimate the carotenoid contents hereafter referred to as tCaro. In addition to the tCaro model, a literature-based index developed for soybean [10] was selected and applied to estimate carotenoid contents from the canopy spectral reflectance data. The carotenoid content calculated using this index is hereafter referred to as iCaro. To calculate iCaro, following equation derived from Chappelle et al [10] was applied to the canopy reflectance measurements from 2009 and 2010.

$$\text{iCaro = [4}{\text{.14 × k × (S}}_{\text{760}}{\text{/S}}_{\text{500}}\text{) - 1.171] × 2}$$ (1)

where S_760_ and S_500_ are the canopy reflectance at 760 nm and 500 nm wavebands, ‘k’ is the reference spectrum constant that represents the mean of the S_500_/S_760_ ratio of all genotypes at a given sampling, 1.171 is the intercept, and 2 is the factor applied to convert concentration (μg mL^-1^) to content (μg cm^2^).

### Descriptive Statistics, BLUP Calculations and Heritability

Descriptive statistics and Pearson correlation analysis were conducted using PROC MEAN and PROC CORR procedures of SAS Version 9.3 (SAS Institute Inc., Cary, NC, USA). To derive phenotypes for genome-wide association mapping, best linear unbiased predictions (BLUPs) values were determined to reduce error variance and shrink the phenotypic values towards the mean [49]. For each phenotype (eCaro, tCaro, and iCaro), data from both years were used to calculate one BLUP value to represent each genotype (S1 Table). BLUPs were determined using the PROC MIXED procedure of SAS [49, 50] as described in [46]. All effects were considered as random for BLUP analysis. Broad sense heritability estimates for eCaro, tCaro and iCaro were derived using variance components obtained from the PROC MIXED procedure of SAS Version 9.3 as described by [51, 52].

### Population Structure analysis

The Bayesian model-based software program STRUCTURE 2.2 [53] was used to infer the population structure of the 332 soybean genotypes based on the 31,253 SNPs with a minor allele frequency (MAF) cut-off of ≥ 5%. The MAF cut-off of ≥ 5% was chosen based on previously published work on soybean [54, 55]. The length of burn-in period and the number of Markov Chain Monte Carlo (MCMC) replications were all assigned at 100,000. The population structure analysis was performed with ten independent iterations with an admixture and allele frequencies correlated model [56]. Thus, in total 100 datasets were obtained with the hypothetical number of subpopulations (*k*) ranging from 1 to 10. The correct estimation of *k* was provided by joining the log probability of data [LnP(D)] from the STRUCTURE output and an *ad hoc* statistic Δ*k* [57], which was based on the rate of change in the log probability of data between successive *k* values based on maximizing log probability or the value at which LnP(D) reached a plateau. Based on the optimum *k* (*k* = 8), each soybean accession was assigned to a subpopulation and the population structure (Q) was generated for further analysis.

### SNP Genotyping and Association Mapping

The genotypic data for the 332 soybean accessions was obtained from the application of the SoySNP50K iSelect SNP Beadchip (S1 Fig) [58]. In total, 31,253 polymorphic SNPs with a MAF ≥ 5% across the 332 genotypes were used for genome-wide association mapping of eCaro, tCaro, and iCaro. Linkage disequilibrium (LD) was calculated using all 31,253 SNP and 332 genotypes. The PLINK software program was used for the calculation of LD (*r* ^*2*^) based upon SNPs within 1 Mb windows [59]. Separate LD calculations were made for euchromatic and heterochromatic chromosomal regions.

Association mapping was conducted based on the BLUP values using a mixed linear model (MLM) with Q-matrix and K-matrix. The Q and K matrices were used as corrections for population structure and/or genetic relatedness [46, 60–63]. The “Analysis/Kinship” submenu function in TASSEL 5.2.3 software was used for generation of the kinship matrix (K). All 31,253 SNPs were used for generation of K, based on scaled Identity by State (IBS) similarity method as described [64]. The Q matrix was generated by STRUCTURE 2.2 [53] software with optimum sub-population structure (*k = 8*) and used along with kinship matrix (K) for association mapping.

Association mapping based on MLM+Q+K model was conducted with TASSEL 5.2.3 [65, 66]. Multiple testing was performed to assess the significance of marker trait associations using QVALUE R 3.1.0 employing the smoother method [67], an extension of the false discovery rate (FDR) method [68]. Markers with *q*FDR < 0.05 were considered significant. All markers that satisfied the multiple testing threshold (*q*FDR < 0.05) had–log10 *P* values ≥ 3.2, which is greater than the threshold (-log10 P values > 3.0) used in other published reports for soybean [54, 69, 70].

## Results

### Environmental Conditions and Carotenoid Content

**Fig 1:**
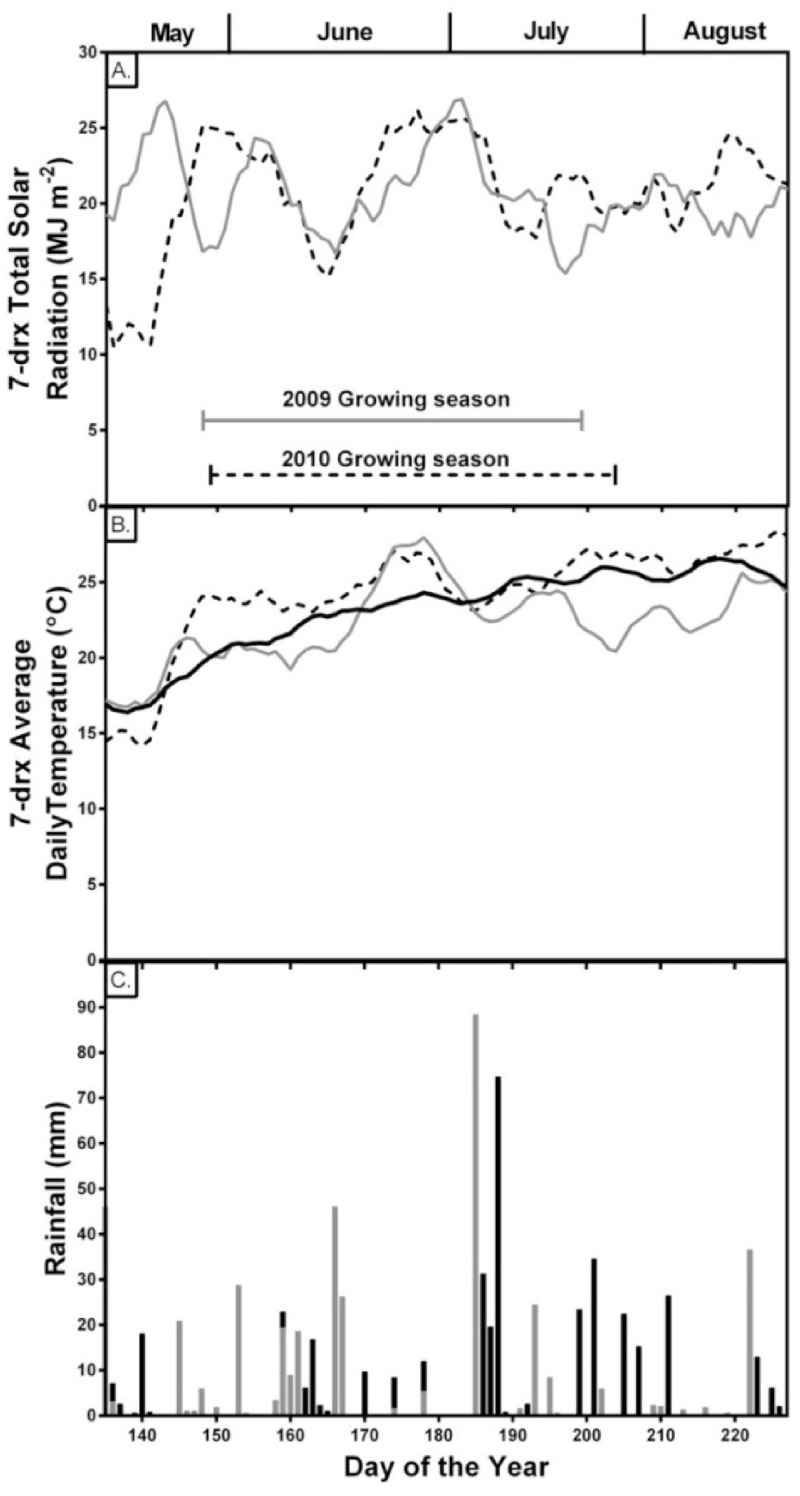
Seven-day running averages versus day of year for solar radiation (A), Average temperature (B), and the daily rainfall (C), during the 2009 and 2010 growing seasons. The solid gray line indicates 2009 and dashed blacked line indicates 2010 growing season. Solid black line indicates the average daily temperature from 2001 to 2010. Solid grey and black bars indicate 2009 and 2010 daily rainfall, respectively.

Distinct differences in precipitation patterns (Fig 1) and cumulative precipitation (2009: 312 mm; 2010: 272 mm) were observed between the two years, but irrigation was not necessary in either year. Although incident solar radiation was similar for the two seasons overall, for a few days immediately before sampling, solar radiation was low in 2009 compared to 2010. For the most part, daily maximum and minimum temperatures between planting and plant sampling for tissue analyses were lower in 2009 than in 2010, averaging 22.9 and 24.7°C, respectively.

**Fig 2:**
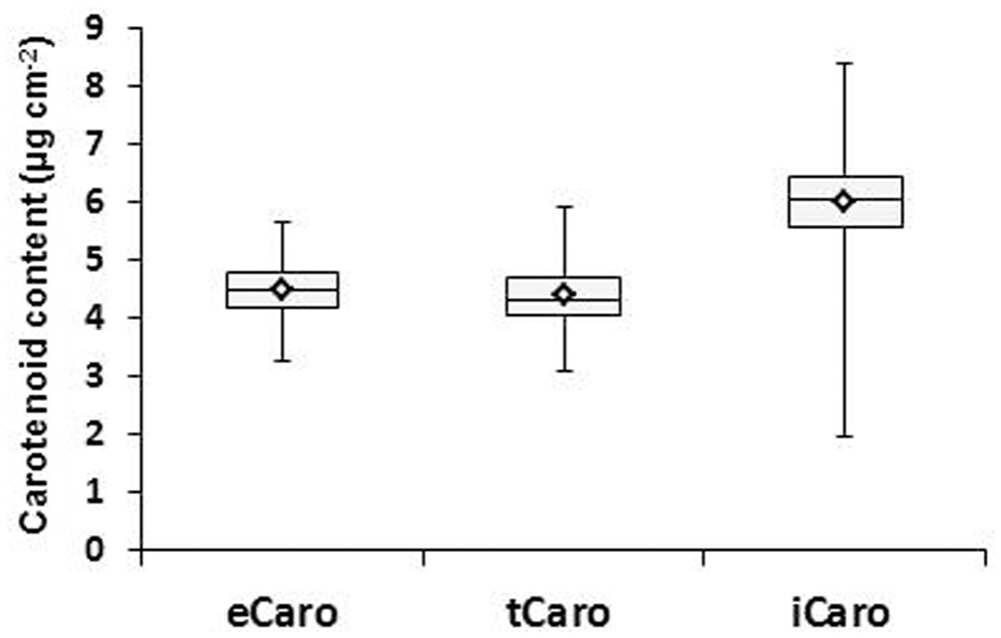
Box plot showing differences in carotenoids across two years (2009 and 2010) using extractable carotenoid content (eCaro), wavelet transformed spectral reflectance carotenoid content (tCaro) and spectral index carotenoid content (iCaro). Box edges represent the upper and lower quartile with median value shown as a bold line near the middle of each box. Mean values are represented by the diamonds and the upper and lower whiskers represent the extreme values.

Analysis of variance revealed significant environment effects for eCaro, tCaro, and iCaro (P<0.0001). However, no genotype by environment interactions was observed. Fig 2 reveals the broad range of carotenoid contents observed among the 332 MG IV soybean genotypes for eCaro, tCaro and iCaro. Mean and median were similar for eCaro and tCaro, and both were considerably smaller than for iCaro. The range in carotenoid contents across the two years was smallest for eCaro (2.36 μg cm^-2^) intermediate for iCaro (2.82 μg cm^-2^) and largest for iCaro (6.40 μg cm^-2^) determinations.

The relationships among carotenoid determination methods were examined by correlation analyses. Significant correlations between eCaro and tCaro (r = 0.42, P<0.001) and between eCaro and iCaro (r = 0.12, P<0.001) were found. In contrast, correlation between tCaro and iCaro was not significant (r = 0.03, P = 0.48). Calculations of broad-sense heritability revealed the highest heritability for iCaro (56.28%) followed by eCaro (38.03%) and tCaro (26.97%).

### Population Structure and LD decay

A model-based approach of population structure analysis was conducted on 332 soybean genotypes with 31,253 SNPs to identify the number of subpopulations (*k*). The results indicated that the optimal number of groups was *k* = 8 (S2 Fig). The eight groups were labeled G1 to G8 as illustrated in S3 Fig. The contributions of genotypes from different geographical regions (countries) varied considerably among groups (S2 Table). The first group, G1, had genotypes exclusively from South Korea (100% for G1). South Korean genotypes were also the majority in G3 (80.55%), G4 (84.40%), and G6 (92.85%). Among groups, G5 was the smallest group with genotypes from South Korea and China represented in equal numbers. In G8, genotypes from South Korea were dominant (58.33%) whereas for G2, genotypes from China were dominant (77.50%). The only group in which genotypes from South Korea or China did not represent the majority was G7 in which genotypes from Japan were dominant (57.37% for G7) (S2 Table).

In this study, LD analysis was performed using the 31,253 SNPs with MAF ≥ 5% and the 332 soybean genotypes evaluated. The LD decay was much higher in the euchromatic compared to heterochromatic regions. In euchromatic regions, the LD decayed half of its maximum value within approximately 85 kb and in heterochromatic regions, the LD did not decay to half of the maximum value within 1 Mb (S4 Fig). These results were consistent with previous results for which the genotypes evaluated in this study were a subset of a larger (373) panel of genotypes [46] as well as for another report for soybean [69].

## Association Mapping

**Fig 3:**
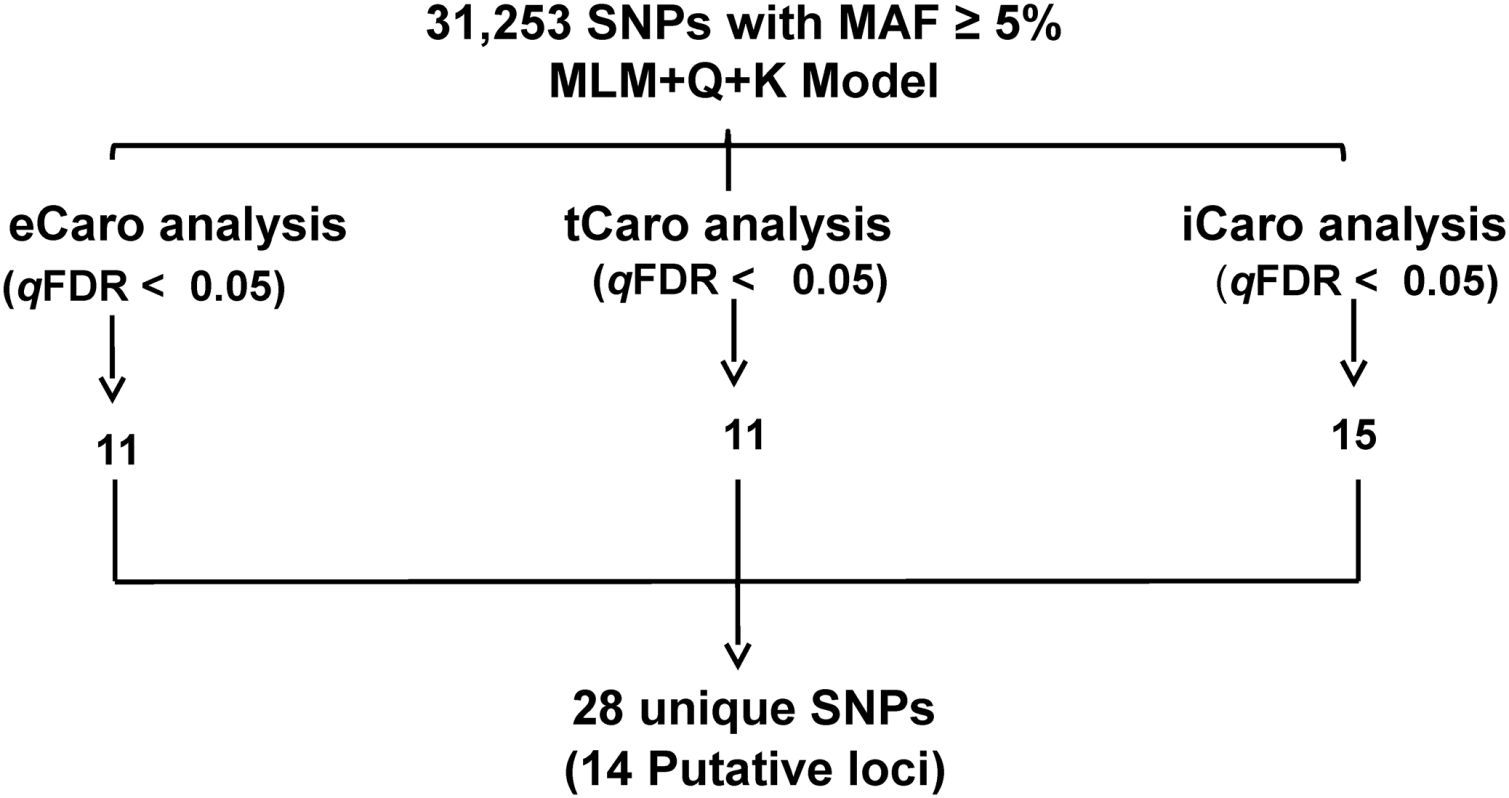
Flow chart showing the SNP selection for extractable carotenoid content (eCaro), wavelet transformed spectral reflectance carotenoid content (tCaro) and spectral index carotenoid content (iCaro) based on MLM+Q+K from the original 31,253 SNPs with MAF ≥ 5% analyzed across two years (2009 and 2010) in Columbia. For all analyses, BLUP means were used for association testing.

Association mapping of 31,253 SNP markers with BLUP values for eCaro, tCaro and iCaro was conducted using MLM+Q+K model. The Q (population structure) and K (kinship) matrices were used as corrections for population structure and/or genetic relatedness and to help avoid false positives [63, 71]. Additionally, SNP associations were evaluated using multiple testing adjustments [67, 68] at a *q*FDR threshold of P < 0.05. The schematic overview of the process employed to reduce the 31,253 SNPs to 28 unique SNPs associated with 14 putative genomic loci for the three carotenoid content measures is shown in Fig 3.

**Fig 4:**
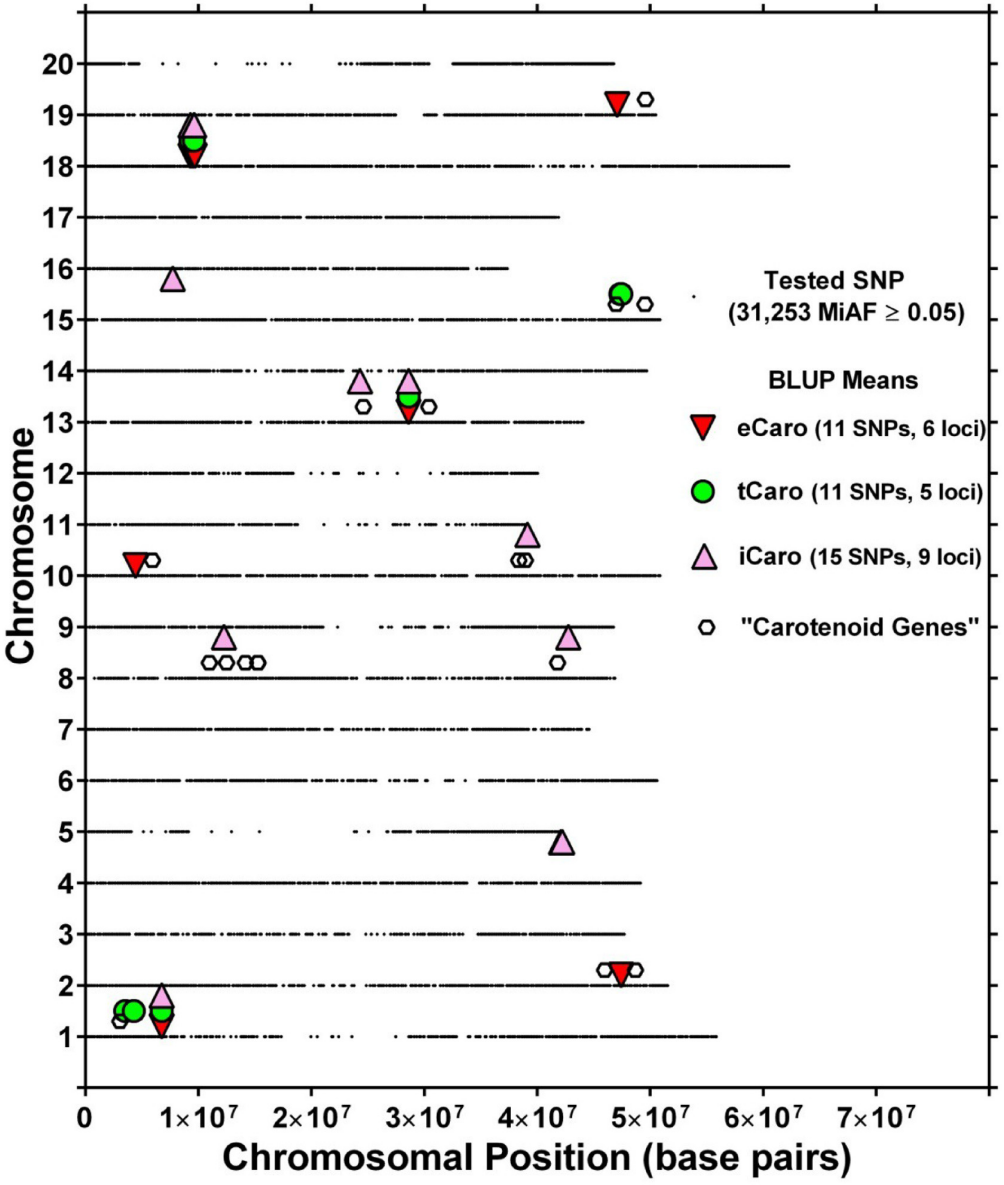
Location of putative loci significantly associated with carotenoids (eCaro, tCaro and iCaro) using MLM+Q+K model and carotenoid-related genes identified in Soybase. For each chromosome, the black dots represent the location of a SNP evaluated. Putative “carotenoid genes” were identified using the search term “carotenoid” in Soybase (www.soybase.org) and were located within ± 3MB of putative loci.

**Fig 5:**
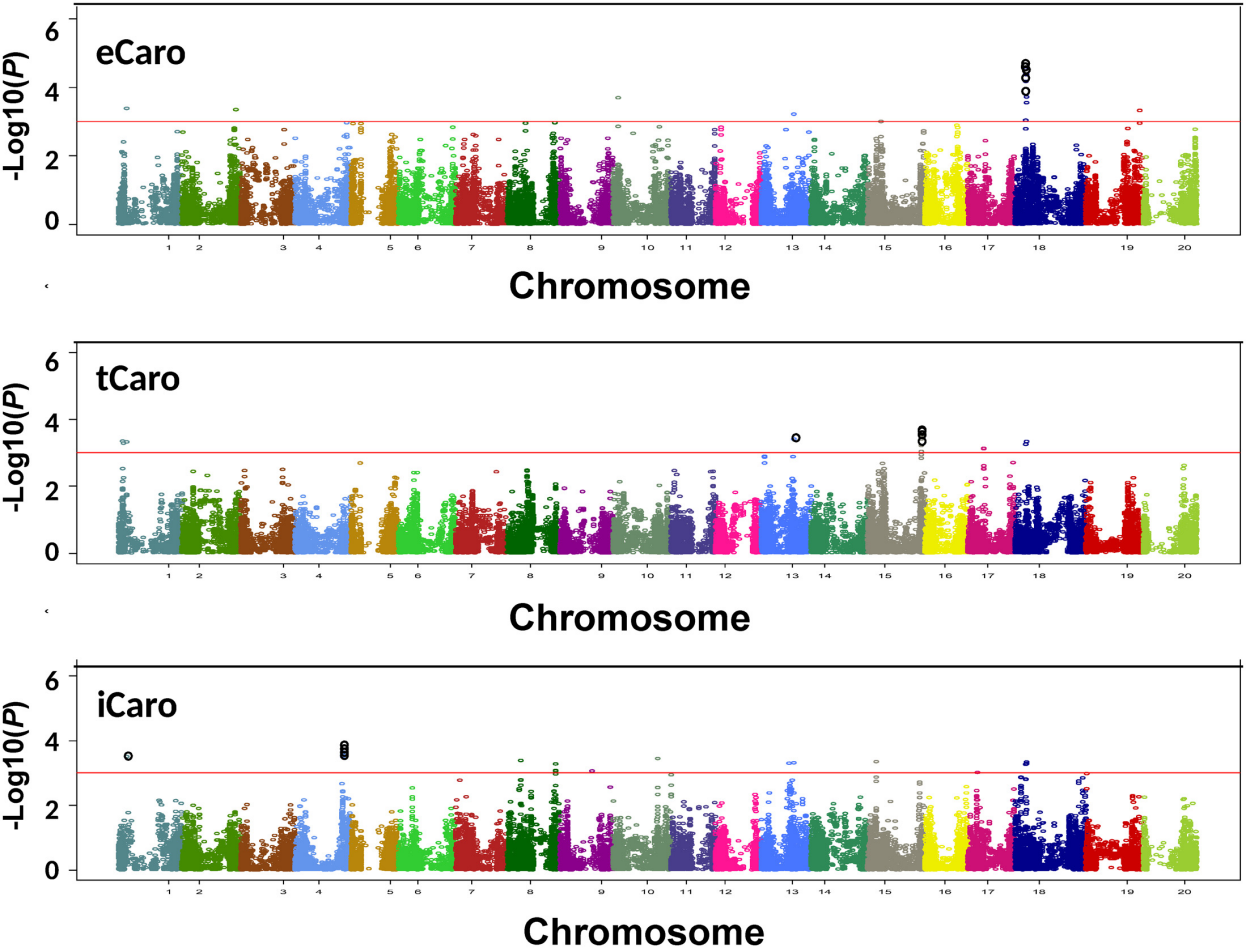
Manhattan plot of-Log10 (*P*) vs. chromosomal position of SNP markers for extractable carotenoid content (eCaro), wavelet transformed spectral reflectance carotenoid content (tCaro) and spectral index carotenoid content (iCaro) using MLM+Q+K model. The red line represents the threshold level of-Log 10*P* ≥ 3.00. The five SNPs that showed the most significant association for each carotenoid phenotype are circled in black.

Association analysis identified a total of 11 SNPs significantly associated with eCaro BLUP values (Fig 3). SNPs in close proximity likely identify the same locus. Thus, the 11 unique SNPs associated with eCaro likely identified six putative loci (loci 2, 3, 7, 10, 13 and 14, Table 1; Fig 4). The putative eCaro locus on chromosome 18 (locus 13, Table 1) was identified by six closely spaced SNPs, and the remaining five loci were each identified by one SNP showing significant association with eCaro (Table 1 and Fig 3). The allele effects for eCaro (percent change in carotenoid content for the major compared to the minor allele) are shown in Table 1 and ranged from -19.34% to 24.81%. For three of the six loci associated with eCaro the minor allele was associated with increased carotenoid content (loci 2, 10, and 13, Table 1) and for three loci the major allele was associated with increased carotenoid content (loci 3, 7, and 14, Table 1). The minor allele was associated with an increase in carotenoid content for all six of the closely spaced SNPs on chromosome 18 (locus 13, Table 1). The five strongest SNP associations with eCaro were located at the putative locus on chromosome 18 (Fig 5 and Table 1).

**Table 1:** List of 28 putative candidate SNPs significantly associated with extractable carotenoid content (eCaro), wavelet transformed spectral reflectance carotenoid content (tCaro) and spectral index carotenoid content (iCaro) using MLM+Q+K model. The 28 SNPs identified 14 putative genomic loci.

Locus	SNP ID	MAF^1^	Major Allele	Minor Allele	-log10 p value	*q*FDR^2^	R2 Value	Allele Effect^3^	Method
1	BARC_1.01_Gm_01_3512849_C_T	0.16	C	T	3.34	0.046	4.11	-16.39	tCaro
	BARC_1.01_Gm_01_4276007_T_C	0.47	T	C	3.28	0.052	4.03	-11.10	tCaro
2	BARC_1.01_Gm_01_6754321_G_A	0.11	G	A	3.37	0.043	2.75	-5.55	eCaro
					3.31	0.048	4.14	-20.61	tCaro
					3.49	0.032	4.30	-28.57	iCaro
3	BARC_1.01_Gm_02_47434930_T_C	0.21	C	T	3.34	0.045	3.45	12.55	eCaro
4	BARC_1.01_Gm_04_42091078_A_G	0.35	G	A	3.52	0.030	4.33	17.95	iCaro
	BARC_1.01_Gm_04_42143026_G_A	0.35	A	G	3.64	0.023	4.50	18.40	iCaro
	BARC_1.01_Gm_04_42153936_T_C	0.36	C	T	3.62	0.024	4.49	18.62	iCaro
	BARC_1.01_Gm_04_42197347_T_C	0.35	C	T	3.49	0.032	4.29	18.06	iCaro
	BARC_1.01_Gm_04_42211808_C_T	0.35	T	C	3.55	0.028	4.53	20.07	iCaro
5	BARC_1.01_Gm_08_12262341_A_G	0.34	A	G	3.38	0.042	3.80	16.69	iCaro
6	BARC_1.01_Gm_08_42740835_A_C	0.19	A	C	3.26	0.055	4.05	-15.56	iCaro
7	BARC_1.01_Gm_10_4416883_T_C	0.41	T	C	3.68	0.021	4.34	24.81	eCaro
8	BARC_1.01_Gm_10_39125336_G_T	0.32	G	T	3.44	0.037	3.84	17.58	iCaro
9	BARC_1.01_Gm_13_24293637_T_C	0.29	T	C	3.29	0.051	4.07	17.59	iCaro
10	BARC_1.01_Gm_13_28592949_G_A	0.12	A	G	3.21	0.046	2.68	-2.68	eCaro
					3.40	0.040	3.19	-13.98	tCaro
					3.30	0.050	3.19	-17.48	iCaro
11	BARC_1.01_Gm_15_7718600_A_G	0.14	A	G	3.34	0.046	4.06	-20.97	iCaro
12	BARC_1.01_Gm_15_47347846_C_T	0.16	C	T	3.40	0.040	4.34	-6.20	tCaro
	BARC_1.01_Gm_15_47355084_C_T	0.15	C	T	3.39	0.040	4.22	-16.97	tCaro
	BARC_1.01_Gm_15_47403548_G_A	0.24	G	A	3.49	0.033	4.33	-13.65	tCaro
	BARC_1.01_Gm_15_47404988_C_T	0.24	C	T	3.45	0.036	4.33	-14.35	tCaro
	BARC_1.01_Gm_15_47421641_T_C	0.27	T	C	3.44	0.036	4.26	-12.97	tCaro
13	BARC_1.01_Gm_18_9284632_A_G	0.21	A	G	4.16	0.007	5.01	-18.23	eCaro
					3.24	0.057	2.95	-1.95	tCaro
					3.25	0.056	4.06	-24.92	iCaro
	BARC_1.01_Gm_18_9433511_C_T	0.21	C	T	4.52	0.003	5.46	-19.34	eCaro
	BARC_1.01_Gm_18_9474722_G_T	0.21	G	T	4.36	0.004	5.32	-17.96	eCaro
	BARC_1.01_Gm_18_9603626_A_C	0.21	A	C	4.36	0.004	5.26	-19.05	eCaro
					3.33	0.047	2.87	-1.45	tCaro
					3.28	0.052	4.05	-23.83	iCaro
	BARC_1.01_Gm_18_9625531_A_G	0.23	A	G	3.71	0.019	3.04	-12.06	eCaro
					3.33	0.047	3.63	-21.50	iCaro
	BARC_1.01_Gm_18_9676741_G_A	0.20	G	A	3.55	0.028	4.11	-18.17	eCaro
14	BARC_1.01_Gm_19_47069443_T_C	0.30	C	T	3.32	0.048	3.49	11.14	eCaro

^1^ Minor allele frequency

^2^ Q-value software derived false discovery rate

^3^ The effect was calculated as the percent change in carotenoid concentrations (major to minor allele).

Using BLUP values for tCaro, a total of 11 SNPs were identified using the MLM+Q+K model (Fig 3). Likely these 11 SNPs identified five putative loci based on their genomic location (loci 1, 2, 10, 12, and 13, Table 1; Fig 4). The putative tCaro locus on chromosome 15 (locus 12, Table 1) was identified by five closely spaced SNPs, and one of two loci on chromosome 1 was identified by two closely spaced SNPs, while the remaining three loci were identified by one SNP (Table 1 and Fig 4). The allele effects for tCaro ranged from -20.61% to -1.45% (Table 1). Thus all SNPs identified as associated with tCaro exhibited an increase in carotenoid content for the minor allele over the major allele. This was consistent for both putative loci for which multiple SNPs associated with tCaro were identified (loci 1 and 12, Table 1). The five strongest SNP associations with tCaro, were all found at the putative locus on chromosome 15 (Fig 5 and Table 1).

Association mapping for iCaro identified 15 SNPs (Fig 3). Together these 15 SNPs likely identified nine putative loci (loci 2, 4, 5, 6, 8, 9, 10, 11, and 13, Table 1; Fig 4). Of the nine putative iCaro loci, one locus on chromosome 4 (locus 4, Table 1) was identified by five closely spaced SNPs and one locus on chromosome 18 (locus 13, Table 1) was identified by three closely spaced SNPs. The remaining seven loci were identified by one SNP each (Table 1 and Fig 4). The allele effect for iCaro (Table 1) ranged from -28.57% to 20.07% with five of the loci (2, 6, 10, 11, and 13, Table 1) associated with increased carotenoid increase for the minor allele over the major allele. For all five of the closely spaced SNPs associated with iCaro at locus 4 (Table 1), there was increased carotenoid content for the major allele over the minor allele whereas for locus 13 (Table 1) all four SNPs associated with iCaro were associated with increased carotenoid content for the minor allele. Four of the five strongest SNPs for iCaro marked the putative locus on chromosome 4, while locus 2 on chromosome 1 was marked by a single SNP (Fig 5 and Table 1).

**Fig 6:**
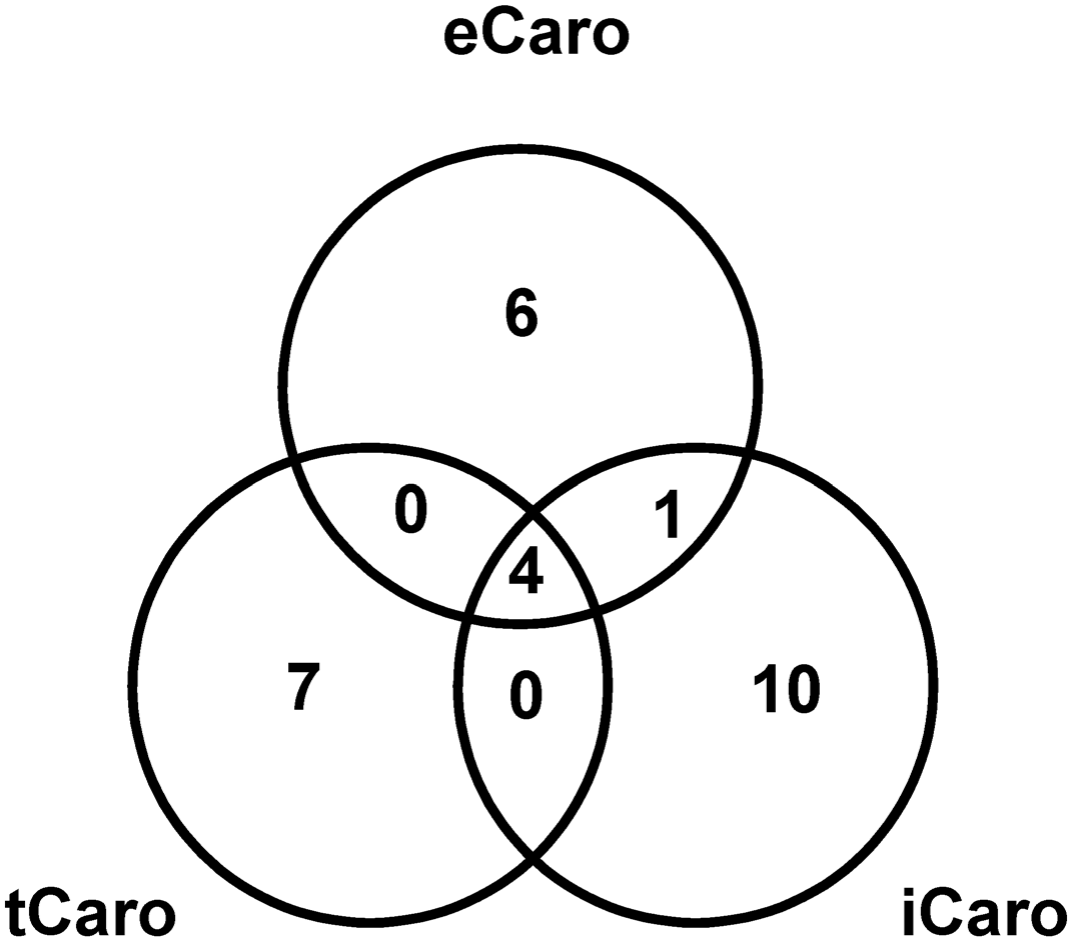
Venn diagram showing number of SNPs significantly associated with extractable carotenoid content (eCaro), wavelet transformed spectral reflectance carotenoid content (tCaro) and spectral index carotenoid content (iCaro) using MLM+Q+K model.

Comparisons of significant SNPs identified for each of the three carotenoid determination methods (eCaro, tCaro and iCaro) revealed four SNPs that were identified based on all three methods as well as one SNP on chromosome 18 was identified by two (eCaro and iCaro) methods (Fig 6). The four SNPs identified by all three methods were part of three putative loci located on chromosomes 1, 13, and 18 (loci 2, 10, and 13, Table 1, Fig 4). The locus on chromosome 18 had two SNPs identified by all three carotenoid measures. Additionally, one other SNP at the locus on chromosome 18 was identified by two methods (eCaro and iCaro; Table 1, Fig 4). In total, 14 putative loci were identified using the three carotenoid determination methods (Fig 4). Three of these were identified using all three carotenoid determination methods and the remaining 11 putative loci were all identified using only one of the three methods of carotenoid determination. Of these 11 putative loci, three were identified by eCaro, two by tCaro, and six by iCaro (Table 1, Fig 4). Interestingly, for each of the three loci identified by all three carotenoid determination methods (loci 2, 10, and 13, Table 1) the allele effects were consistent in that higher carotenoid content was associated with the minor allele.

### Identification of Putative Candidate SNPs and Genes

Based on 60 bp sequences flanking the 28 candidate SNPs (Table 1), a blast search was conducted with default parameters in Soybase (www.soybase.org) [72] to identify putative candidate genes, but none of these genes have any obvious functional relationship with carotenoid content. An additional search for candidate genes was performed in Soybase using the term “carotenoid.” This search revealed 76 genes, 19 of which were located within ± 3 MB of one of the 28 unique candidate SNPs (Table 2, Fig 4). Six of these 19 genes were near an eCaro putative locus, four near a tCaro putative locus, and 11 were near an iCaro putative locus. A total of 10 of the 14 putative loci had at least one carotenoid related gene nearby. Among these was one of the three putative loci (chromosome 13, Fig 4) identified using all three carotenoid determination methods (Glyma13g27220, Table 2). Within the ± 3 MB range, four of the six loci identified by eCaro, three of the five loci identified by tCaro, and five of the nine loci identified by iCaro had a likely known carotenoid-related gene within ± 3 MB (Fig 4).

**Table 2:** List of 19 known carotenoid-related genes within a ± 3MB region of the 28 putative candidate SNPs identified as significantly associated with carotenoid content. Genes were identified in Soybase (www.soybase.org) using the search term “carotenoid”.

Name of Gene^1^	Start	Stop	Source^2^	Functional annotation	eCaro^3^	tCaro	iCaro
Glyma01g03530	3043844	3047833	Soybase	ATP citrate synthase	No	Yes	No
Glyma02g40720	45947276	45952779	Soybase	Squalene/phytoene synthase	Yes	No	No
Glyma02g43993	48680221	48689596	KEGG pathway	Abscisic-aldehyde oxidase [EC:1.2.3.14]	Yes	No	No
Glyma08g15115	10958559	10961561	Soybase	Violaxanthin de-epoxidase [EC:1.10.99.3]	Yes	No	No
Glyma08g17010	12476922	12481872	Soybase	GTP-specific succinyl-CoA synthetase, beta subunit	No	No	Yes
Glyma08g18801	14154786	14157329	Soybase	9-cis-epoxycarotenoid dioxygenase [EC:1.13.11.51]	No	No	Yes
Glyma08g20190	15241495	15246946	Soybase	Oxidation/reduction process (Lipooxygenase activity)	No	No	Yes
Glyma08g20210	15264345	15270063	Soybase	Oxidation/reduction process (Lipooxygenase activity)	No	No	Yes
Glyma08g20220	15275016	15280833	Soybase	Oxidation/reduction process (Lipooxygenase activity)	No	No	Yes
Glyma08g20230	15305056	15308538	Soybase	Oxidation/reduction process (Lipooxygenase activity)	No	No	Yes
Glyma08g41890	41781278	41784567	Soybase/KEGG pathway	Biosynthetic process (Squalene/phytoene synthase)	No	No	Yes
Glyma10g07210	5912252	5918724	Soybase/KEGG pathway	Cytochrome P450 CYP4/CYP19/CYP26 subfamilies	Yes	No	No
Glyma10g29490	38350166	38356416	Soybase	Oxidation/reduction process (Lipooxygenase activity)	No	No	Yes
Glyma10g30220	38926992	38932155	Soybase/KEGG pathway	Zeta-carotene desaturase [EC:1.14.99.30]	No	No	Yes
Glyma13g21110	24588817	24595787	Soybase/KEGG pathway	Cytochrome P450 CYP4/CYP19/CYP26 subfamilies	No	No	Yes
Glyma13g27220	30386135	30392625	Soybase	9-cis-epoxycarotenoid dioxygenase [EC:1.13.11.51]	Yes	Yes	Yes
Glyma15g40070	46990056	46991412	Soybase	9-cis-epoxycarotenoid dioxygenase [EC:1.13.11.51]	No	Yes	No
Glyma15g42140	49529893	49534600	Soybase	ATP citrate synthase	No	Yes	No
Glyma19g44010	49571464	49574835	Soybase/KEGG pathway	Violaxanthin de-epoxidase [EC:1.10.99.3]	Yes	No	No

^1^Name of Gene is based on information in Soybase

^2^ Soybase and/or KEGG carotenoid biosynthetic pathway

^3^ Yes or No indicates the presence of gene in ± 3Mb of putative SNPs identified by respective carotenoid determination methods

## Discussion

### Carotenoid Contents

Broad ranges of carotenoid contents were observed among the 332 soybean genotypes for all three determination methods (Fig 2). The eCaro and tCaro average values observed were similar to each other and to carotenoid contents reported previously for soybean [73, 74]. Since [37] used the eCaro values from the 332 genotypes examined in the present study to arrive at the model that was used in this study to determine the tCaro values, this was expected. In comparison, while derived from the same canopy spectral reflectance measurements as the tCaro values, iCaro determinations were based on a completely independent index developed for soybean by [10]. Thus, it is not surprising that iCaro values are not as closely related to eCaro values as the tCaro phenotype. Nonetheless, the range of iCaro encompasses all observed eCaro and tCaro carotenoid contents. Since iCaro values were determined based on canopy-level reflectance, the reflectance spectrum characteristics represent leaves differing in age and relative position in the canopy. In contrast, for the determination of the eCaro phenotypes leaf disks were collected from uppermost fully expanded, sun-exposed leaflets. Given the difference in sampling area, a broader range of iCaro than eCaro phenotypes among the 332 genotypes was expected as leaf age and position are known to influence carotenoid content [75–78]. Correlation analyses among eCaro, tCaro, and iCaro revealed a significant relationship between eCaro and tCaro and eCaro and iCaro combinations. The lack of correlation between iCaro and tCaro is interesting in that both of these traits are based on the same spectral reflectance measurements, albeit using different wavelength signatures for the calculation of carotenoid content. Nonetheless, as discussed below, association analysis revealed multiple SNPs that were in common among all three carotenoid phenotypes as well as between one of the three possible two-way combinations (Fig 6).

### Population Structure

Understanding genetic relationships and the population structure of the germplasm evaluated is critical to control false positives in association mapping [79]. Soybean population structure has been well studied using both SSR and SNP markers for *Glycine max* and *Glycine soja* [46, 61, 69, 80]. The estimated population structure of the 332 accessions evaluated in this study indicated few subpopulations exhibiting distinctive identities. The accessions were classified into eight subpopulations with significant divergence among subpopulations. Similar results were observed in previous studies using 373 soybean genotypes with 12,347 SNP markers and 31,145 SNP markers [46, 61].

### Association mapping of eCaro, tCaro and iCaro

Association mapping facilitates the detection and mapping of quantitative trait loci (QTLs) underlying complex traits in the absence of bi-parental populations. In the present study, application of *q*FDR <0.05 drastically reduced the number of markers from several thousand to 15 or fewer, depending on the carotenoid trait (Fig 3). A greater number of significant SNP associations were identified using iCaro (15) followed by eCaro (11) and tCaro (11) using MLM+Q+K model for all three carotenoid determination methods employed.

It is important to note that the three putative loci significantly associated with all three carotenoid content traits were found on chromosomes 1, 13 and 18. The identification of identical SNP associations for more than one carotenoid phenotype is of particular interest, suggesting these markers to be very robust and increasing confidence in these associations.

### Putative Loci and Potential Candidate Gene Identification

Twenty eight unique SNPs were identified to be the most promising candidates for their association with soybean leaf/canopy carotenoid content (Fig 3, Table 1). A search for carotenoid related genes in Soybase revealed 19 genes in the vicinity (± 3MB) of these 28 SNPs (Table 2). The chromosomal locations of the 28 SNPs and 19 potential candidate genes are illustrated in Fig 4. Likely these SNPs indicate 14 putative loci in nine chromosomal regions. Three putative loci were identified by SNPs significantly associated with all three carotenoid phenotypes and thus, may represent major QTLs. Of these three putative loci, one locus on chromosome 13 was located near a gene encoding the carotenoid cleavage enzyme 9-cis-epoxycarotenoid dioxygenase [EC: 1.13.11.51] an enzyme that is involved in carotenoid cleavage and important for ABA biosynthesis [81, 82] (Table 2, Fig 4). Coupled with the documented function of the proteins encoded by these genes, the detection of loci based on all three carotenoid traits in their vicinity, suggests an important role in the determination of soybean leaf (eCaro) and canopy carotenoid contents (iCaro and tCaro). No known carotenoid gene was found near the two other putative loci (chromosome 1 and 18) that were identified using all three carotenoid determination methods. However, since these putative loci were discovered based on all three methods, they may represent previously unknown genes that modulate carotenoid contents. Clearly these putative loci are candidates for greater research focus.

Of the five loci with the largest increases in carotenoid content associated with a minor allele, two (loci 10 and 12, Table 1) were near genes annotated as 9-cis-epoxycarotenoid dioxygenase [EC: 1.13.11.51], locus 10 being the one on chromosome 13 that was identified by all three methods. A more thorough examination of the two putative 9-cis-epoxycarotenoid dioxygenase [EC: 1.13.11.51] genes may provide previously unknown genetic variation associated with carotenoid content in soybean.

Two of five loci with the largest increases associated with higher carotenoid content of the major allele were located on chromosome 10 (loci 7 and 8; 24.81% and 17.58% respectively, Table 1 and Fig 4). Each locus was tagged by one SNP with locus 7 identified based on eCaro and locus 8 based on iCaro (Table 1). Locus 7 was near a gene-related to a Cytochrome P450 CYP4/CYP19/CYP26 subfamily protein which was also found near locus 9 (Table 1). The protein sequences of these two Cytochrome P450 genes have >82% similarity with a cytochrome P450 monoxygenase protein (LUT1), that has been shown to play an important role in lutein production in *Arabidopsis* (Tian et al., 2004). Locus 8 was near two putative carotenoid related genes, one identified as zeta-carotene desaturase [EC:1.14.99.30] and the other as lipoxygenase (Table 1). The zeta-carotene desaturase gene is found in the Kyoto Encyclopedia of Genes and Genomes (KEGG) pathway of carotenoid metabolism in cereals [83]. Interestingly, one SNP (locus 5 on chromosome 8) had seven carotenoid related genes nearby (violaxanthin de-epoxidase [EC:1.10.99.3], GTP-specific succinyl-CoA synthetase, 9-cis-epoxycarotenoid dioxygenase [EC:1.13.11.51] and four genes with lipooxygenase activity). No putative carotenoid genes were found near the other locus with a large effect (locus 4 on chromosome 4; Table 1) The remaining loci without putative carotenoid genes in their vicinity may be associated with new genes and may be promising targets for further investigations (locus 2, 4, 11 and 13).

Putative loci identified by SNPs based on one carotenoid phenotype were located on chromosomes 1, 2, 4, 8, 10, 13, 15 and 19 and may represent minor QTLs. It is notable that one of the two loci identified based on the iCaro phenotype on chromosome 8 had seven carotenoid related genes nearby, and two carotenoid related genes were located near loci on chromosomes 2 (eCaro) and 15 (tCaro), and one of the loci on chromosome 10 (iCaro) (Fig 4). The proximity of several carotenoid related genes near loci identified based on single carotenoid phenotypes (eCaro, tCaro, or iCaro) provides added confidence that these loci are true positives for carotenoid content. Nonetheless, we have greater confidence in the 3 loci identified based on all three carotenoid determination methods. Genes or regulatory factors in the vicinity of these putative loci are expected to be important in determining leaf and/or canopy carotenoid content of field-grown soybean.

### Use of Canopy Spectral Reflectance for Association Mapping

Canopy spectral reflectance characteristics can be assessed rapidly and non-destructively and are used for numerous purposes. In this study, two methods (tCaro and iCaro) were used to determine canopy carotenoid content based on the same canopy spectral reflectance measurements. Genome-wide association analysis using these two methods resulted in the identification of nine putative loci for iCaro and five putative loci for tCaro, including three loci that were identified for both (Fig 4). Fourteen genes annotated as carotenoid-related are located in the vicinity of the loci identified based on one or both of these phenotypes (Table 2). This, together with the identification of a subset of SNPs that were identical for these two phenotypes and the eCaro phenotype, indicates that canopy spectral reflectance characteristics can be used to map leaf and canopy carotenoid contents in soybean. The significant overlap of markers identified based on iCaro and those identified by tCaro and eCaro also indicates the robustness of the index developed by [10], and suggests that at least some literature-based indices may be used to identify genetic markers based on canopy spectral reflectance. Further, these results demonstrate the feasibility of coupling field-based, high-throughput canopy spectral reflectance phenotyping with genomic data to identify genetic loci associated with plant canopy traits.

## Conclusions

Genome-wide association mapping using a mixed linear model (MLM+Q+K) resulted in the identification of 28 SNPs putatively associated with soybean leaf and canopy carotenoid contents. These SNPs likely represented 14 putative loci associated with three different measures of carotenoid content of 332 soybean genotypes. The fact that these putative loci were identified based on data from two distinct growing seasons provides added confidence in their accuracy and reliability. Candidate loci identified based on canopy spectral reflectance characteristics (tCaro, iCaro) indicate that markers for canopy carotenoid contents can be identified and that high-throughput phenotyping based on canopy spectral reflectance can provide useful phenotypes for association mapping.

## Supporting Information

S1 Fig Steps to retrieve genetic SNP marker data from SoyBase (www.soybase.org) for the 332 genotypes evaluated. (PDF)

S2 Fig Population structure results using 31,253 SNPs. Log probability data LnP(D) as function of *k* (number of groups) from the structure run. The plateau of the graph at *k* = 8 indicates the optimum number of subgroups possible in the panel.
(PPTX)

S3 Fig Model-based population structure of 332 soybean genotypes (*k* = 8). The y-axis is the subgroup membership, and the x-axis is the individual genotypes in each sub population (G1–G8). All 31,253 markers with MAF ≥5% was used for analysis.
(PPTX)

S4 Fig Extent of LD decay in euchromatic (a) and heterochromatic region (b) with a 1 Mb window and using 31,253 SNPs. (PPTX)

S1 Table BLUP means of carotenoid content estimated by three methods of carotenoid determination over 332 genotypes. The three methods were extractable carotenoid contents (eCaro), wavelet transformed spectral reflectance carotenoid contents (tCaro), and spectral index carotenoid contents (iCaro).
(XLSX)

S2 Table The origin distribution of 332 genotype arranged by eight subgroups (G1–G8) determined by model-based STRUCTURE analysis of 31,253 SNPs. (DOCX)
