# Supplementary material for: Association Mapping of Total Carotenoids in Diverse Soybean Genotypes Based on Leaf Extracts and High-Throughput Canopy Spectral Reflectance Measurements
Source: PLoS One. 2015 Sep 14;10(9):e0137213. doi: 10.1371/journal.pone.0137213 (PMC4569184; doi:10.1371/journal.pone.0137213)
Supplement: S1 Fig — (PDF) [file pone.0137213.s001.pdf]

S1 Fig. Steps to retrieve genetic SNP marker data from SoyBase (www.soybase.org) for the 332 genotypes evaluated.

1. SoySNP50K iSelect SNP BeadChip data across 16,652 G.max and G.soja accessions genotypes with 54,041 SNPs is freely available from Soybase (www.soybase.org). Go to Soybase (www.soybase.org) home page and move the cursor over Soybase (highlighted in yellow as below)

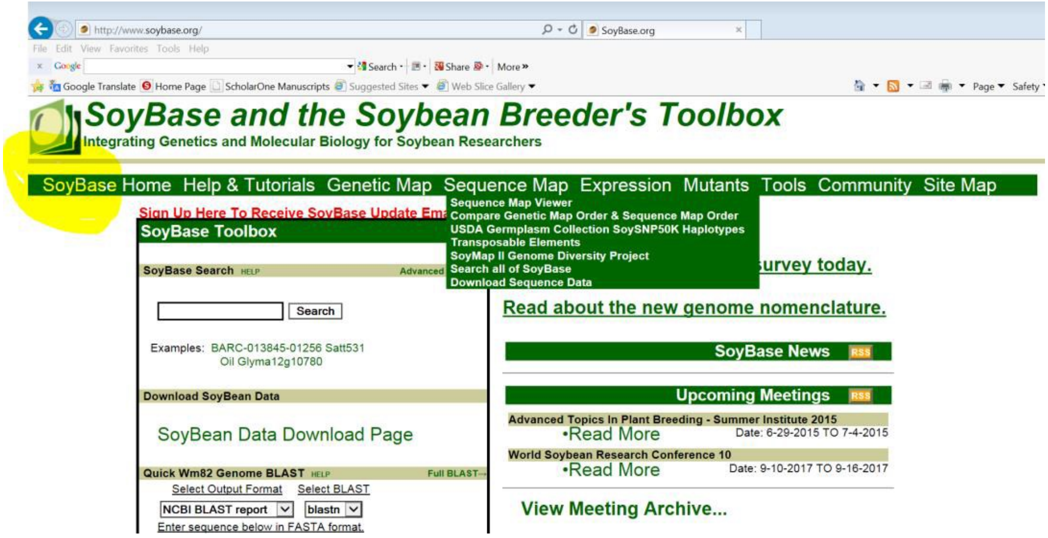

2. In the drop down menu, select the second choice: "Download Sequence and Genetic Data"

3. In the new pop-up window scroll to bottom where it says "Download SoySNP50K data"

Download SoySNP50K Data

The SoySNP50K iSelect BeadChip has been used to genotype the USDA Soybean Germplasm Collection (Song et al. in preparation) and the data generously provided by the authors before publication.

The complete data set for 19,652 G. max and G. soja accessions genotyped with 52,041 SNPs is available here (approx. 170 Mb compressed with GZIP) .

SoySNP50K haplotypes for a user-selected subset of the genotyped cultivars can be downloaded from this page.

4. First option is to download the complete data set of 19, 652 G.max and G. soja accessions genotyped with 52, 041 SNPs.

5. Second option is to download the user-selected subset of accessions (for eg. 332 genotypes)
