# Supplementary material for: Association Mapping of Total Carotenoids in Diverse Soybean Genotypes Based on Leaf Extracts and High-Throughput Canopy Spectral Reflectance Measurements
Source: PLoS One. 2015 Sep 14;10(9):e0137213. doi: 10.1371/journal.pone.0137213 (PMC4569184; doi:10.1371/journal.pone.0137213)
Supplement: S2 Fig — The plateau of the graph at k = 8 indicates the optimum number of subgroups possible in the panel. (PPTX) [file pone.0137213.s002.pptx]

## Slide 1
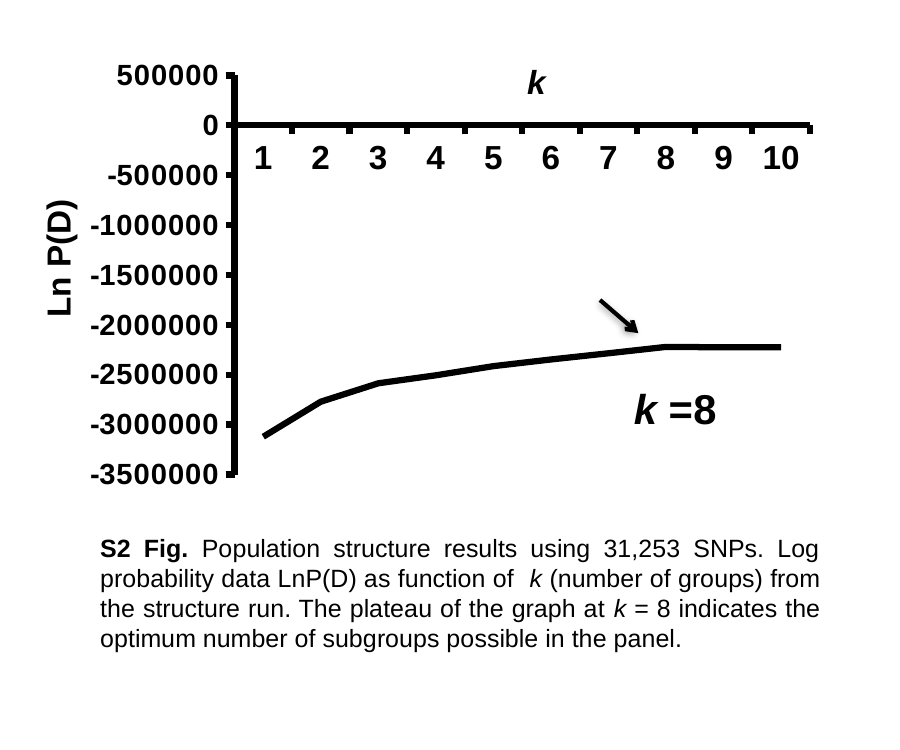

### Chart
| Category | | |
|---|---|---|k =8
k
Ln P(D)
S2 Fig. Population structure results using 31,253 SNPs. Log probability data LnP(D) as function of k (number of groups) from the structure run. The plateau of the graph at k = 8 indicates the optimum number of subgroups possible in the panel.
