# Supplementary material for: Association Mapping of Total Carotenoids in Diverse Soybean Genotypes Based on Leaf Extracts and High-Throughput Canopy Spectral Reflectance Measurements
Source: PLoS One. 2015 Sep 14;10(9):e0137213. doi: 10.1371/journal.pone.0137213 (PMC4569184; doi:10.1371/journal.pone.0137213)
Supplement: S3 Fig — The y-axis is the subgroup membership, and the x-axis is the individual genotypes in each sub population (G1–G8). All 31,253 markers with MAF ≥5% was used for analysis. (PPTX) [file pone.0137213.s003.pptx]

## Slide 1
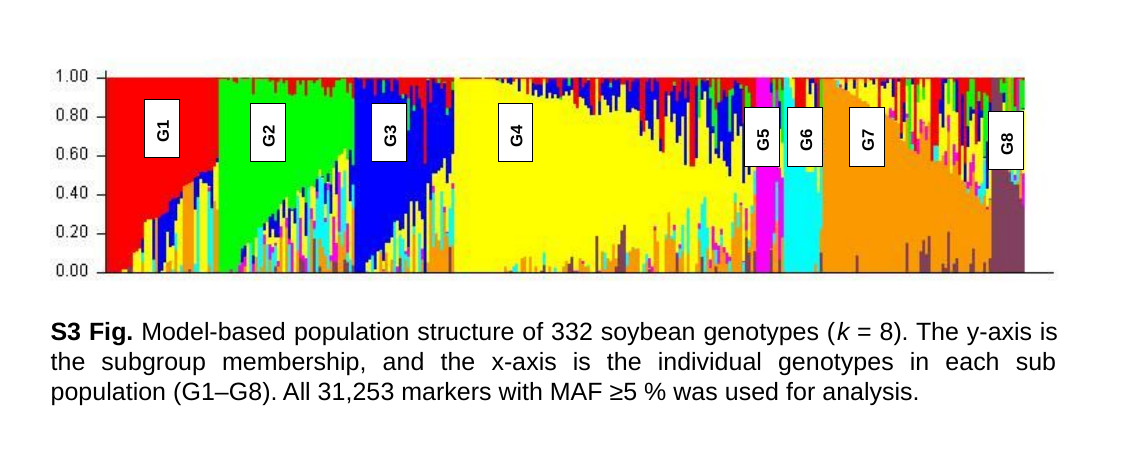

G1
G2
G3
G4
G6
G5
G7
G8
S3 Fig. Model-based population structure of 332 soybean genotypes (k = 8). The y-axis is the subgroup membership, and the x-axis is the individual genotypes in each sub population (G1–G8). All 31,253 markers with MAF ≥5 % was used for analysis.
