# Supplementary material for: Association Mapping of Total Carotenoids in Diverse Soybean Genotypes Based on Leaf Extracts and High-Throughput Canopy Spectral Reflectance Measurements
Source: PLoS One. 2015 Sep 14;10(9):e0137213. doi: 10.1371/journal.pone.0137213 (PMC4569184; doi:10.1371/journal.pone.0137213)
Supplement: S4 Fig — (PPTX) [file pone.0137213.s004.pptx]

## Slide 1
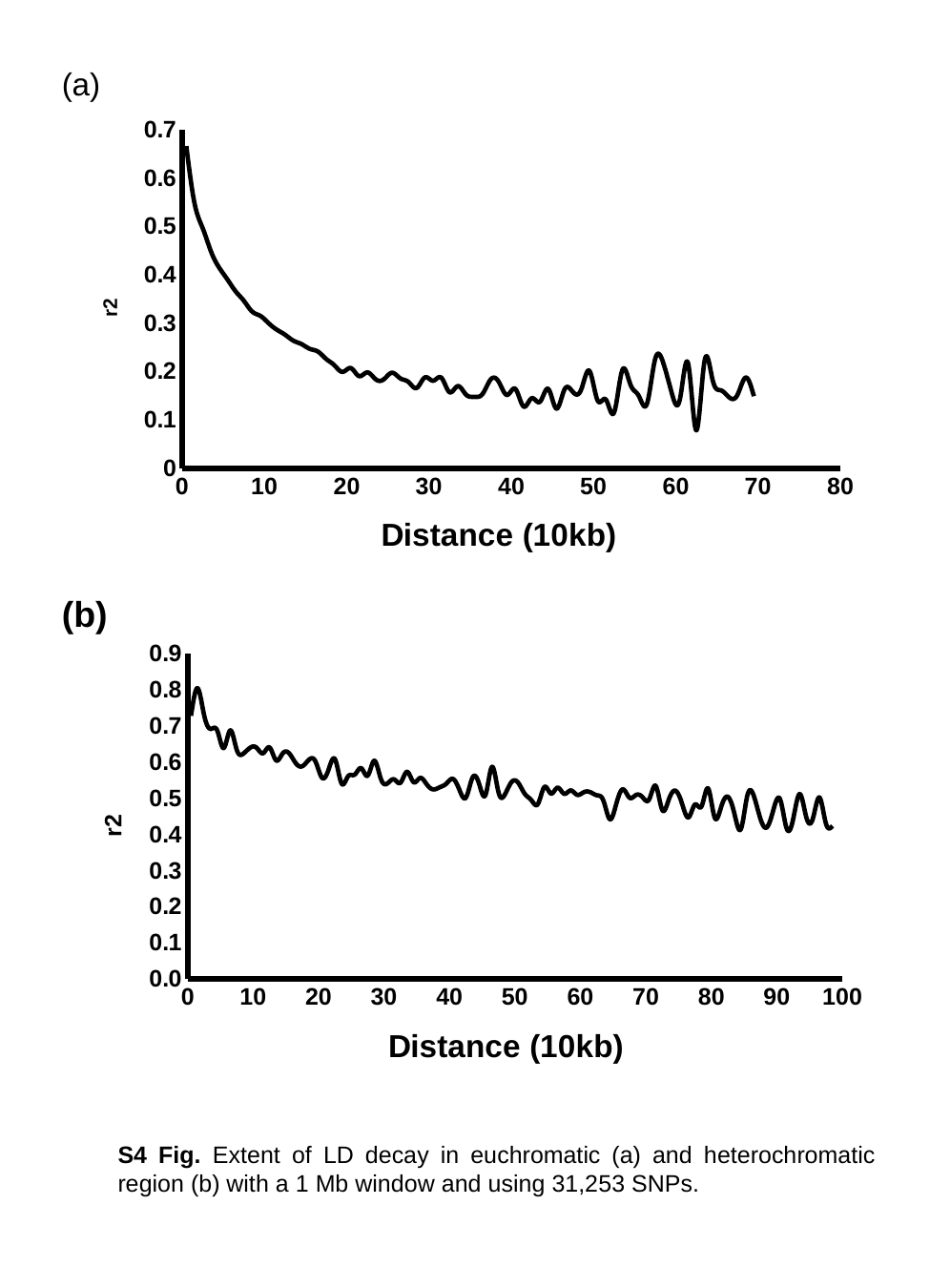

(a)
### Chart
| Category | r2 |
|---|---|(b)
### Chart
| Category | r2 |
|---|---|S4 Fig. Extent of LD decay in euchromatic (a) and heterochromatic region (b) with a 1 Mb window and using 31,253 SNPs.
