# Supplementary material for: Association Mapping of Total Carotenoids in Diverse Soybean Genotypes Based on Leaf Extracts and High-Throughput Canopy Spectral Reflectance Measurements
Source: PLoS One. 2015 Sep 14;10(9):e0137213. doi: 10.1371/journal.pone.0137213 (PMC4569184; doi:10.1371/journal.pone.0137213)
Supplement: S2 Table — (DOCX) [file pone.0137213.s006.docx]

**S2** **Table** The origin distribution of 332 genotype arranged by eight subgroups (G1-G8) determined by model-based STRUCTURE analysis of 31,253 SNPs

| **Subpopulation groups** | **Number of genotypes** | **Distribution of genotype origin** |
| --- | --- | --- |
| G1 | 41 | 41 South Korea (100%) |
| G2 | 49 | 38 China (77.5%); 4 Georgia (8.16); 3 South Korea (6.12%); 2 North Korea (4.08%); 1 Russia (2.01%); 1 Taiwan (2.04); |
| G3 | 36 | 29 South Korea (80.55%); 4 North Korea (11.11%); 1 Korea (2.77%); 1 Japan (2.77%); 1 China (2.77%) |
| G4 | 109 | 92 South Korea (84.40%); 8 China (1.33%); 3 Korea (2.75%); 2 North Korea (1.83%); 2 Japan (1.83%); 1 Romania (0.91%); 1 India (0.91%); |
| G5 | 10 | 4 South Korea (40%); 4 China (40%); 1 Mexico (10%); 1 North Korea (10%) |
| G6 | 14 | 13 South Korea (92.85%); 1 China (7.15%) |
| G7 | 61 | 35 Japan (57.37%); 17 South Korea (27.86%); 3 China (4.91%); 2 Georgia (3.27); 2 North Korea (3.27%); 1 Russia (1.63%); 1 Taiwan (1.63%) |
| G8 | 12 | 7 South Korea (58.33%); 4 China (33.33%); 1 Japan (8.33%); |
